# Artificial intelligence, parasites and parasitic diseases

**DOI:** 10.1186/s13071-023-05972-1

**Published:** 2023-09-28

**Authors:** Filipe Dantas-Torres

**Affiliations:** Aggeu Magalhães Institute, Oswaldo Cruz Foundation (Fiocruz), Recife, PE Brazil

Artificial intelligence (AI) gained renewed attention last year with the release of ChatGPT (OpenAI), an AI-powered language model that uses deep learning techniques to generate human-like text [1]. This revolutionary tool presents many opportunities as well as challenges in the field of scientific writing such as, for example, speeding up the writing and increasing the overall quality of texts (e.g. spelling, grammar, usage and style) [2, 3]. Overall, these advantages are particularly useful for non-native English speakers, thus increasing inclusiveness [4]. However, concern has been expressed regarding the impact AI-generated texts on data reporting, plagiarism and fabrication [2–5]. ChatGPT has been even listed as a co-author in some articles, a development with which many scientists and publishers disagree as AI-text generators do not meet the standard or criteria for authorship [6], particularly in terms of accountability for the work reported.

Indeed, when writing a scientific article, the author needs to elaborate a proper rationale for the investigated hypothesis based on the available literature, which may be available in different languages. Can current AI-text generators elaborate a proper rationale based on a balanced account of the literature by simultaneously assessing multi-language, regional electronic databases? Possibly not, and this could ultimately help to increase—rather than decrease—inequalities in science.

Scientific articles are not only about spelling, style, usage and grammar. There are several other aspects pertaining to scientific writing that may vary widely according to journal style, discipline and target readership. Specialized journals may require a writing style that may not be acceptable for more generalist journals, and vice versa. Similarly, writing styles may vary widely across disciplines and research fields. Classic examples include articles reporting modeling studies (e.g. [7]), clinical trials (e.g. [8]) or descriptions of new parasite species (e.g. [9]), which are very different in terms of both form and content.

*Style* aside, scientific writing is an integral part of science and is the defining moment when the author writes down all details of the work conducted, also sharing experiences that were acquired in the laboratory, field or both. Regarding sharing, I remember the first article I wrote with my friend Professor Domenico Otranto (University of Bari), back in 2008. We were in Buenos Aires for the Sixth Tick and Tick-Borne Pathogen Conference. Long story short, we had a good time writing a review article, while enjoying tea together at the restaurant of his hotel. The resulting article was so long that it had to be divided into two parts [10, 11]. Looking back to this and other moments shared with colleagues and friends while writing scientific articles in different parts of the globe, I would never think about asking an AI-text generator to write these articles for me. These are unique moments that help researchers to develop their writing skills, to share professional and personal experiences, as well as to come up with new ideas and concepts for addition projects and articles. We, as authors, should not lose our humanity, which derives from the culture and personal history of each of us. Definitively, this is something AI can never simulate enough.

While the use of AI to generate scientific articles has been an issue of debate, AI and machine learning methods (e.g. deep learning) have been successfully used in various research fields, including prospection of new antimalarial candidates [12], mosquito species identification [13], diagnosis of parasitic infections [14] and the mining of Apicomplexa dense granule protein-coding genes in genomic datasets [15]. The potential of AI as a research tool in parasitology is tremendous. In this context, *Parasites and Vectors* has just launched the new article collection *Artificial intelligence, parasites and parasitic diseases*. This collection is dedicated to articles reporting the use of AI in parasitology research. I particularly welcome articles dealing with parasitic disease diagnosis, parasite and vector identification as well as those reporting the prospection of drugs and vaccine candidates. If you have a review proposal, please do not hesitate to contact me.

This collection will serve as a platform for authors to publish their research that employs AI and deep learning methods for solving research questions in the fields of parasitology and tropical medicine.
